# Clinical Presentation and Outcomes of Acute Pericarditis in a Large Urban Hospital in the United States of America

**DOI:** 10.1016/j.chest.2020.07.039

**Published:** 2020-07-24

**Authors:** Alessandra Vecchié, Juan G. Chiabrando, Megan S. Dell, Aldo Bonaventura, Adolfo G. Mauro, George Wohlford, Benjamin W. Van Tassell, Daniel H. Berrocal, Fabrizio Montecucco, Anna Beutler, John F. Paolini, Tamas S. Gal, Antonio Abbate

**Affiliations:** a Pauley Heart Center, Virginia Commonwealth University, Richmond, VA; b First Clinic of Internal Medicine, Department of Internal Medicine, University of Genoa, Genoa, Italy; c Interventional Cardiology Department, Hospital Italiano of Buenos Aires, Buenos Aires, Argentina; d Department of Biostatistics, Virginia Commonwealth University, Richmond, VA; e Department of Pharmacotherapy and Outcome Sciences, Virginia Commonwealth University, Richmond, VA; f Kiniksa Pharmaceuticals Ltd., Lexington, MA; g IRCCS Ospedale Policlinico San Martino Genova—Italian Cardiovascular Network, Genoa, Italy; h First Clinic of Internal Medicine, Department of Internal Medicine and Centre of Excellence for Biomedical Research (CEBR), University of Genoa, Genoa, Italy

**Keywords:** acute pericarditis, recurrence, subacute presentation, therapy failure, troponin I, AMI, acute myocardial infarction, ESC, European Society of Cardiology, ICD, International Statistical Classification of Diseases and Related Health Problems

## Abstract

**Background:**

Acute pericarditis is the most common presentation of pericardial diseases. Although generally benign, complications such as constrictive pericarditis, cardiac tamponade, and recurrence can occur.

**Research Question:**

What are the clinical factors associated with adverse outcomes in acute pericarditis?

**Study Design and Methods:**

We used an informatics-based search engine to search for International Classification of Diseases codes related to pericardial disease between January 1, 2009 and November 14, 2018 and then extracted clinical information, including only patients meeting the European Society of Cardiology criteria for acute pericarditis. We then evaluated the predictive value of clinical characteristics for adverse outcomes (cardiac tamponade, constrictive pericarditis, failure of therapy, recurrences, or death).

**Results:**

We identified 240 patients with a first episode of pericarditis (51 [34-62] years, 56% males and 50% white). Pericarditis was determined to be idiopathic in 126 (53%) cases and related to cardiac injury in 79 (33%). During a median follow-up time of 179 (20-450) days, 82 (34%) patients experienced at least one adverse outcome. Subacute presentation was an independent predictor of adverse outcomes. Patients with postcardiac injury pericarditis had a lower incidence in the composite of failure of treatment and recurrence (13% vs 26%; *P* = .022) compared with patients with idiopathic pericarditis. Troponin I measurements were obtained in 167 patients (70%). Elevated troponin I levels were associated with lower incidence of recurrences (4% vs 17%; *P* = .024) and of the composite outcome (13% vs 36%; *P* = .004).

**Interpretation:**

Acute pericarditis is associated with at least one adverse outcome in 34% of patients. Subacute presentation and idiopathic etiology are associated with higher incidence of adverse outcomes, whereas elevated troponin I levels identify a group with reduced risk of recurrences.

FOR EDITORIAL COMMENT, SEE PAGE 2262

Acute pericarditis is the most common presentation of pericardial diseases affecting subjects of both sexes and represents an important cause of morbidity, especially in patients experiencing recurrence.^1^^,^^2^ Epidemiological data are scarce, possibly because of the low sensitivity of diagnostic methods. Previous observational studies have reported a prevalence of acute pericarditis of 27.7 cases per 100,000 person-years in an urban area of Northern Italy.^3^ A large epidemiologic study conducted in Finland reported an incidence of 4.52 per 100,000 person-years for men and 2.11 per 100,000 person-years for women.^4^ TB is the most common cause in low-income countries,^5^ and idiopathic pericarditis is the most reported cause in developed countries. However, postcardiac injury syndrome is a growing cause of pericarditis, likely because of an increase in the number of interventional procedures and cardiac surgeries.^6^ Idiopathic pericarditis has a poorly studied pathophysiology^7^ and is considered to be the result of auto-inflammation possibly related to an immune response to viral infections.^8^^,^^9^

Patients who have a favorable response to antiinflammatory drugs generally have a good prognosis. However, complications such as cardiac tamponade, constrictive pericarditis, and episodes of recurrence may occur.^7^^,^^8^ In addition, adverse drug reactions are common in the currently recommended treatment regimens.^10^, ^11^, ^12^, ^13^ In an attempt to risk stratify patients with chest pain, cardiac biomarkers (ie, troponin T and I) are routinely measured. The prognostic role of these cardiac biomarkers in pericarditis is, however, less well established.^14^^,^^15^

We aimed to analyze the clinical characteristics of patients presenting with acute pericarditis at the Virginia Commonwealth University Medical Center (Richmond, VA), a large urban hospital in the United States, and to gain insights into clinical features associated with an increased risk of adverse outcomes, such as recurrences, constrictive pericarditis, and cardiac tamponade.

## Patients and Methods

### Study Design

We conducted a retrospective review employing an informatics engine-based search (TriNetX).^16^ TriNetX is a global federated network of health research, providing access to data from electronic medical records of patients in large health-care organizations in the United States.^17^ For the initial subject identification, the International Statistical Classification of Diseases and Related Health Problems (ICD)-10 code related to acute pericarditis diagnosis (I30) was used. TriNetX automatically includes the corresponding ICD-9 codes. We identified 494 adult patients between January 1, 2009 and November 14, 2018, and two experienced physicians performed a chart review through electronic health records to complete a database including 240 patients meeting the European Society of Cardiology (ESC) criteria for acute pericarditis (e-Fig 1).^1^ According to guidelines, the diagnosis of acute pericarditis requires at least two of the four following criteria: (1) pericardial chest pain; (2) pericardial rubs; (3) new ST-elevation or PR depression on ECG; (4) new or worsening pericardial effusion.^1^ Inpatients and outpatients were both included. The database contained relevant demographic data, clinical characteristics, treatments received, and adverse outcomes. All data, including ECG and echocardiographic data, were reviewed by at least two cardiologists before entry into the final database. Disagreements on findings or readings were resolved through discussion. The study complied with the Declaration of Helsinki. The chart review was approved by the local Institutional Review Board.

### Study End points and Definitions

The primary goal was to identify predictors of the composite adverse outcome (tamponade, constrictive pericarditis, failure of treatment, clinical recurrences, or death) in a large cohort of patients with acute pericarditis. Secondary aims were to provide an overview of the pericarditis causes and to compare clinical and laboratory profiles among subgroups (eg, with or without adverse outcomes).

Patients were identified as having an acute event if they met the 2015 ESC guideline criteria for acute pericarditis.^1^ A recurrent episode requires new signs and symptoms of pericardial inflammation after a symptom-free interval of 4 to 6 weeks.^1^ Subacute pericarditis was defined as an event developing in an interval from 6 weeks to 3 months.^1^ Constrictive pericarditis was defined by the development of hemodynamic diastolic impairment due to a chronic inflammation or thickening of the pericardium diagnosed at echocardiography, cardiac magnetic resonance, or surgical biopsy. Tamponade was identified as a pericardial effusion, resulting in hemodynamic compromise, diagnosed with echocardiographic criteria.^1^ We considered failure of treatment as the persistence of symptoms regardless of antiinflammatory treatment for >4-6 weeks.^1^ A severe pericardial effusion during an echocardiogram was defined as the presence of an echo-free space >20 mm according to the classification of Imazio et al^18^ and Weitzman et al.^19^ Heart failure at presentation was defined as having signs and symptoms of volume overload, or the need to initiate treatment with a loop diuretic. Meeting any of the following constituted the composite end point of adverse outcomes (“composite outcome”): failure of treatment, recurrence of pericarditis, cardiac tamponade, constrictive pericarditis, or death.

### Statistical Analysis

Continuous data were reported as median and interquartile range, and data were compared with the Mann-Whitney *U* test. Categorical variables were expressed as numbers and percentages and compared using χ^2^ test or Fisher exact test, as appropriate.

In light of the current, poor knowledge about clinical variables that are predictive of adverse outcomes in acute pericarditis, especially in a heterogeneous population such as the one of this study, we have chosen clinically relevant variables to be put in a logistic univariate model and tested for their statistical significance. Those meeting statistical significance in the univariate model (*P* < .1, two-tailed) were incorporated in the multivariate logistic regression model to identify independent variables associated with the composite outcome. For the univariate and multivariate analysis, the measure of uncertainty was expressed as an OR and 95% CI. All statistical analyses were performed using IBM SPSS Statistics for Windows, Version 25.0 for Mac (IBM).

## Results

### Clinical Characteristics of the Patients

**Figure 1:**
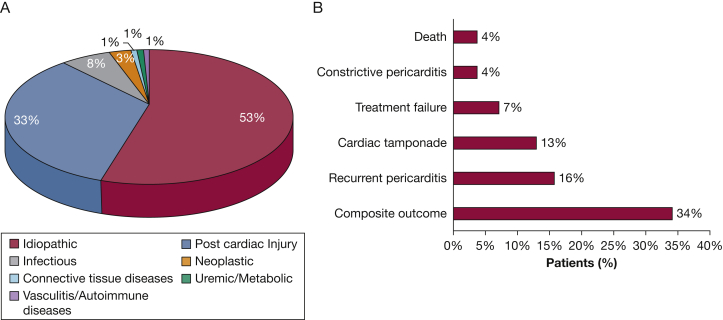
A, Etiology of acute pericarditis. The pie chart shows the prevalence of various acute pericarditis etiologies in the cohort. B, Adverse outcomes. The column chart shows the incidence of adverse outcomes in the cohort.

A total of 240 patients were found to meet acute pericarditis criteria, and the characteristics at the time of presentation are summarized in Table 1. The median age of the cohort was 51 (34-62) years, with 135 (56%) men, and 121 (50.4%) self-defined whites and 100 (41.7%) blacks. Two hundred twenty (92%) patients had pericardial chest pain, 43 (18%) pericardial rubs, 107 (47%) pericardial effusion, and 128 (53%) ECG alterations (71 [30%] PR depression and 104 [43%] ST elevation). Idiopathic pericarditis was found to be the most common diagnosis (126, [52.5%]). Postcardiac injury was the second most frequent cause of pericarditis (79 [32.9%]; Fig 1A). Among patients with postcardiac injury pericarditis, four (5%) had a chest trauma, 11 (14%) an acute myocardial infarction (AMI), 32 (41%) a catheter ablation, and one (1%) thoracoscopic ablation, six (8%) a pacemaker implantation, 11 (14%) surgery for valve repair or replacement, and four (5%) intervention for left appendage exclusion. Eleven (14%) patients had a cardiac bypass surgery, of which three happened after AMI. Ten (13%) patients underwent a percutaneous coronary intervention, eight of which occurred after AMI.

**Table 1:** Characteristics of the Overall Cohort

Characteristics	Overall Cohort (n = 240)
Demographics	
Male sex	135 (56)
Age, y	51 [34-62]
Race	
White	121 (50.4)
Black	100 (41.7)
Other^a^	19 (7.9)
BMI	28 [24-32]
Medical history	
Hypertension	37 (15.4)
Diabetes	51 (21.3)
Dyslipidemia	79 (32.9)
Coronary artery disease	52 (21.7)
Chronic heart failure	51 (21.3)
Stroke/transient ischemic attack	13 (5.4)
Atrial fibrillation	63 (26.3)
Myocardial infarction	33 (13.8)
Active smokers	77 (32.2)
Autoimmune diseases	24 (10.0)
TB	2 (0.8)
Chest radiation	8 (3.3)
Neoplastic diseases	30 (12.5)
Severe chronic kidney disease	44 (18.4)
Chest trauma	4 (1.7)
Recent cardiac procedure	
Percutaneous coronary intervention	7 (2.9)
Pacemaker/cardiac ablation	24 (10.0)
Cardiac surgery	19 (7.9)
Therapies	
Immunosuppression	15 (6.3)
High dose corticosteroids	9 (3.8)
Oral anticoagulation	27 (11.3)
Etiology	
Idiopathic	126 (52.5)
Postcardiac injury	79 (32.9)
Other	35 (14.5)
Clinical presentation	
Fever	21 (8.9)
Subacute presentation	12 (5.1)
Trauma	5 (2.1)
Pericardial effusion	107 (47%)
Severe pericardial effusion	49 (20.6)
Heart failure	43 (18.1)
Chest pain	220 (93.2)
Cardiac examination	
Pericardial rub	43 (18.5)
Pulsus paradoxus	10 (4.3)
Kussmaul sign	4 (1.7)
ECG	
PR depression	70 (30.6)
ST elevation	104 (45.4)
T wave inversion	57 (24.9)
Laboratory	
WBC, No. ×10^3^/mL	9.4 [6.7-12.3]
Neutrophils, No. ×10^3^/mL	6.7 [4.2-9.5]
Lymphocyte, No. ×10^3^/mL	1.6 [1.0-2.0]
Hemoglobin, g/dL	12.1 [10.4-13.5]
Platelets, No. ×10^3^/mL	240 [177-325]
Creatinine, mg/dL	0.89 [0.72-1.1]
C-reactive protein, mg/dL	10.0 [1.6-18.6]
Erythrocyte sedimentation rate, mm/h	52 [23-88]
Troponin ≥ 0.20 ng/mL	46 (27.5)
Treatment at presentation	
Nonsteroidal antiinflammatory drugs	169 (70.7)
Colchicine	152 (63.6)
Glucocorticoids	29 (12.2)
Surgical procedure	
Pericardiectomy	8 (3.3)
Pericardial window	7 (2.9)
Pericardiocentesis	27 (11.3)
Outcomes	
Composite outcome^b^	82 (34.2)
Treatment failure	17 (7.2)
Recurrent pericarditis	38 (15.8)
Cardiac tamponade	31 (13.0)
Constrictive pericarditis	9 (3.8)
Death	9 (3.8)
Rehospitalization for any cause	90 (37.7)

The data are presented as No. (%) of all cases or as median [interquartile range].

a Other includes Asian, Native American, Hispanic, or not specified.

b Composite end point defined as the presence of any of the following: failure of treatment, recurrences, cardiac tamponade, constrictive pericarditis, or death.

Patients presented with a history of cardiovascular disease (chronic heart failure, atrial fibrillation, myocardial infarction, hypertension) in 35% of cases, diabetes in 20%, and autoimmune disease in 10%, with approximately 20% having recently undergone a cardiac procedure.

A small number of patients (12 [5.1%]) had a subacute presentation. A total of 49 patients (20.6%) were found to have a severe pericardial effusion, and 31 (13%) had tamponade.

Laboratory tests showed abnormal inflammatory biomarkers (C-reactive protein, 10 [1.6-18.6] mg/dL; and erythrocyte sedimentation rate, 52 [23-88] mm/h) for those with data available (n = 72, 30%; and n = 61, 25.4%, respectively).

A troponin I measurement during the first pericarditis episode was available for 167 (70%) individuals. When comparing patients with and without troponin I determination, differences were found in the frequency of recent cardiac procedures and ECG alterations, suggesting that troponin was measured to aid in the diagnosis or exclude myocardial injury (e-Table 1). Of the 167 patients who had troponin I measurements at arrival, 46 (27.5%) had elevation in serum troponin I levels (≥ 0.20 ng/mL).

Nonsteroidal antiinflammatory drugs (NSAIDs) were used in 169 (70.7%) cases, colchicine in 152 (63.6%), and glucocorticoids in 29 (12.2%). A total of 189 (80.8%) patients were hospitalized at the time of diagnosis, and 42 (17.5%) required an invasive procedure related to the pericardial disease (pericardiocentesis [n = 27, 11.3%], pericardiectomy [n = 8, 3.3%], or pericardial window [n = 7, 2.9%]).

### Adverse Outcomes

The cohort had a median follow-up time at our institution of 179 (20-450) days after the qualifying episode, and 82 (34.2%) patients experienced at least one of the following adverse outcomes: 38 (15.8%) developed recurrent pericarditis, 31 (13%) tamponade, nine (3.8%) constrictive pericarditis, 17 (7.2%) failure of treatment, and nine (3.8) died (Fig 1B). Rehospitalization for any reason occurred in 90 subjects (37.7%).

No significant differences in baseline demographic characteristics or pericarditis etiology at qualifying event were found when comparing those with and without adverse outcomes (Table 2). Subjects with adverse outcomes were more likely to have had a subacute presentation (12.2% vs 1.3%; OR, 10.7; 95% CI, 2.3-50.1; *P =* .001). Among those with troponin I sampling, subjects who did not experience any adverse outcome presented more frequently with elevated levels of the biomarker (25.3% vs 7.3%; OR, .27; 95% CI, 0.11-0.69; *P* = .004). In addition, patients without adverse outcomes were also more likely to have been prescribed colchicine (69% vs 52.4%; OR, 0.51; 95% CI, 0.29-0.88; *P* = .023). No significant difference was found in the nonsteroidal antiinflammatory drugs or glucocorticoids prescription rates.

**Table 2:** Characteristics of the Overall Cohort Based on the Occurrence of Adverse Outcomes (Tamponade, Constrictive Pericarditis, Failure of Treatment, Clinical Recurrences, or Death)

Characteristics	Uncomplicated Cases (n = 158)	Patients With Adverse Outcome (n = 82)
Demographics		
Male sex	95 (50.1)	40 (48.8)
Age, y	51 [35-62]	53 [32-65]
Race		
White	74 (46.8)	47 (57.3)
Black	70 (44.9)	29 (35.4)
Other	13 (8.2)	6 (7.3)
BMI	28 [24-32]	28 [23-34]
Medical history		
Hypertension	81 (51.3)	45 (54.9)
Diabetes	37 (23.4)	14 (17.1)
Dyslipidemia	51 (32.3)	28 (34.1)
Coronary artery disease	36 (22.8)	16 (19.5)
Chronic heart failure	32 (20.3)	19 (23.2)
Stroke/transient ischemic attack	9 (5.7)	4 (4.9)
Atrial fibrillation	43 (27.2)	20 (24.4)
Previous myocardial infarction	27 (17.1)	6 (7.3)
Active smokers	55 (34.8)	22 (26.8)
Autoimmune diseases	12 (7.6)	12 (14.6)
TB	2 (1.3)	…
Chest radiation	5 (3.2)	3 (3.7)
Neoplastic diseases	15 (9.5)	15 (18.3)
Severe chronic kidney disease	23 (14.6)	21 (25.6)
Chest trauma	4 (2.5)	2 (2.4)
Recent cardiac procedure		
Percutaneous coronary intervention	5 (3.2)	2 (2.4)
Pacemaker/cardiac ablation	17 (10.8)	7 (8.5)
Cardiac surgery	13 (8.2)	6 (7.3)
Therapies		
Immunosuppression	9 (5.7)	6 (7.3)
High-dose corticosteroids	4 (2.5)	5 (6.1)
Oral anticoagulation	24 (15.2)	3 (3.7)
Etiology		
Idiopathic	82 (49.7)	44 (58.7)
Postcardiac injury	61 (37.0)	18 (24.0)
Other	22 (13.3)	13 (10.6)
Clinical presentation		
Fever	15 (9.5)	6 (7.3)
Subacute presentation	2 (1.3)	10 (12.2)
Trauma	4 (2.5)	5 (6.1)
Severe (>20 mm) pericardial effusion	14 (8.9)	35 (42.7)
Heart failure	27 (17.1)	16 (19.5)
Chest pain	150 (94.9)	70 (85.4)
Cardiac examination		
Pericardial rub	31 (19.6)	12 (14.6)
Pulsus paradoxus	3 (1.9)	7 (8.5)
Kussmaul sign	3 (1.9)	4 (4.9)
ECG		
PR depression	48 (30.4)	23 (28)
ST elevation	73 (46.2)	31 (37.8)
T wave inversion	41 (25.9)	17 (20.7)
Laboratory		
WBC, No. ×10^3^/mL	9.0 [6.5-12.5]	9.7 [7.3-11.9]
Neutrophils, No. ×10^3^/mL	6.65 [4.13-9.8]	7.10 [4.45-8.95]
Lymphocyte, No. ×10^3^/mL	1.65 [1-2.3]	1.3 [1-1.9]
Neutrophil/lymphocyte ratio	3.97 [2.31-7.07]	4.80 [3.22-7.98]
Hemoglobin, g/dL	12.4 [10.7-14]	11.4 [9-13.1]
Platelets, No. ×10^3^/mL	233 [167-306]	284 [188-388]
Creatinine, mg/dL	0.89 [0.74-1.08]	0.86 [0.69-1.31]
C-reactive protein, mg/dL	7.8 [1-5-15.3]	14.3 [2.1-21.8]
Erythrocyte sedimentation rate, mm/h	43 [20-83]	71 [42-105]
Troponin ≥ 0.20 ng/mL	40 (25.3)	6 (7.3)
Treatment at presentation		
Nonsteroidal antiinflammatory drugs	116 (73.4)	53 (64.6)
Colchicine	109 (69.0)	43 (52.4)
Glucocorticoids	16 (10.1)	13 (15.9)
Surgical procedure		
Pericardiectomy	2 (1.3)	6 (7.3)
Pericardial window	1 (0.6)	6 (7.3)
Pericardiocentesis	3 (1.9)	24 (29.3)

The data are presented as No. (%) of all cases or as median [interquartile range].

**Figure 2:**
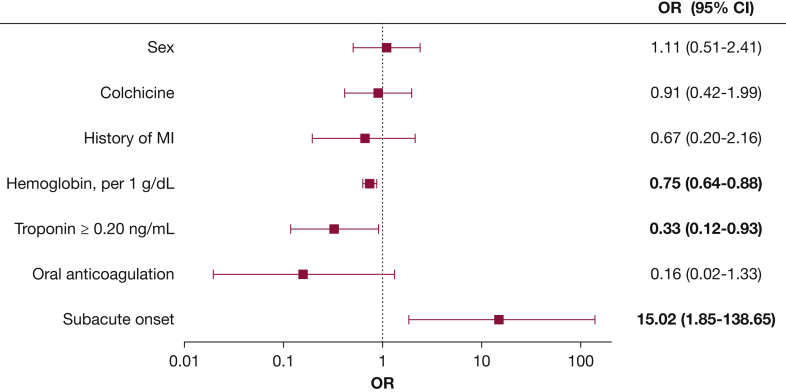
Forest plot for the prediction of the composite adverse outcome. Logistic regression multivariate model for the prediction of the composite adverse outcome (tamponade, constrictive pericarditis, failure of treatment, clinical recurrences, or death). Statistical significant values are presented in bold character. MI = myocardial infarction.

In a logistic regression model, elevated troponin I levels were found to be predictive of lower incidence of the composite outcome during the follow-up (OR, 0.27; 95% CI, 0.11-0.69; *P* = .006), even after adjustment for history of myocardial infarction, oral anticoagulation, subacute presentation, hemoglobin value, and treatment with colchicine (adjusted OR, 0.33; 95% CI, 0.12-0.93; *P* = .035) (Fig 2, e-Table 2). Subacute presentation was found to be predictive for the composite adverse outcome (unadjusted OR, 10.69; 95% CI, 2.28-50.07; *P* = .003, and adjusted OR, 15.02; 95% CI, 1.85-138.65; *P* = .013) (Fig 2, e-Table 2).

### Comparison Between Idiopathic and Postcardiac Injury Pericarditis

**Figure 3:**
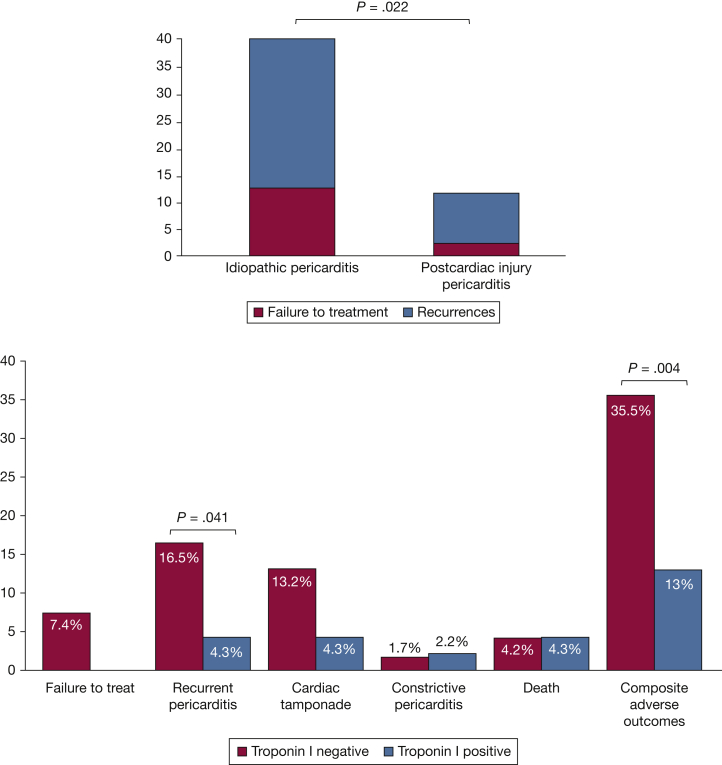
A, Treatment failure or clinical recurrences in idiopathic and postcardiac injury pericarditis. The column chart shows the incidence of treatment failure or clinical recurrences in patients with idiopathic pericarditis and postcardiac injury pericarditis. The column chart shows the incidence of treatment failure or clinical recurrences in patients with idiopathic pericarditis and postcardiac injury pericarditis. B, Adverse outcome in patients with and without troponin I positivity. The column chart shows the incidence of adverse outcome in patients with and without troponin I positivity.

When compared with patients with idiopathic pericarditis, those with postcardiac injury pericarditis were older (61 [50-71] vs 48 [34-58] years; *P* < .001, e-Table 3). A significantly greater number of blacks were found in the idiopathic pericarditis group compared with the postcardiac injury group (70 [55.6%] vs 14 [17.7%]; *P* < .001). The prevalence of other cardiovascular diseases was higher in patients with postcardiac injury pericarditis who also showed a higher prevalence of heart failure at presentation compared with idiopathic pericarditis patients (20 [25.3%] vs 13 [10.3%]; *P* = .006). A higher number of patients with postcardiac injury had elevation in troponin I levels (22 [27.8%] vs 9 [7.1%]; *P* < .001). Finally, there was a higher incidence of the combined failure of treatment or clinical recurrences in patients with idiopathic pericarditis compared with those with postcardiac injury (33 [26.2%] vs 10 [12.7%]; OR 2.44 [1.13-5.30]; *P* = .022) (Fig 3A).

### Troponin I Level as Prognostic Marker

Subjects with elevated troponin I (≥0.20 ng/mL) were more likely to have a history of other cardiovascular diseases (e-Table 4). Patients with abnormal troponin I values also presented more frequently with heart failure symptoms at the onset of pericarditis (14 [30.4] vs 18 [14.9]; *P* = .028), experienced fewer recurrent events (2 [4.3%] vs 20 [16.5%]; OR, 0.23; 95% CI, 0.05-1.03; *P* = .041) and had a reduced occurrence of the composite adverse outcome (6 [13%] vs 43 [35.5%]; OR, 0.27; 95% CI, 0.11-0.69; *P* = .004) (Fig 3B).

## Discussion

Acute pericarditis is a relatively common, often unrecognized clinical condition that has significant complications.^2^ Although several large-scale epidemiologic studies on the incidence and prevalence have been conducted,^3^^,^^4^ a detailed characterization of the condition and its complications is lacking. In the current study, including a mixed population of whites and blacks served at an urban hospital in the United States, we found that (1) subacute presentation is independently associated with a greater risk for the development of adverse outcomes; (2) patients with idiopathic pericarditis more frequently experience treatment failure or clinical recurrences, when compared with postcardiac injury pericarditis; (3) for patients with troponin I obtained, those with elevated troponin I levels were less likely to experience adverse outcomes, such as clinical recurrences.

Subacute, or delayed-onset, presentation had already been reported as an unfavorable prognostic factor for the development of adverse outcomes in acute pericarditis.^1^^,^^18^ The early recognition of acute pericarditis may be important, because timely treatment may reduce both acute inflammatory symptoms and the incidence of recurrence.^20^ A delay in treatment due to a subacute presentation may consolidate the inflammation of the pericardium and markedly increase the complication rate. The definition of subacute presentation, however, remains imprecise and without a clear determination of the time window. Subacute presentation also may be a surrogate for a specific cause of pericarditis that is not identified in these studies, that has a more subtle clinical course yet a higher risk of recurrence. Further prospective clinical studies may help better define appropriate time windows for the pathophysiology and identification of risk factors.

Hemoglobin has been found as a protective factor in our cohort. This finding is novel and unreported in the literature. Inflammation is known to reduce the activity of the bone marrow,^21^ and absence of anemia may be considered as a marker of a lesser degree of inflammation associated with a better prognosis. For postcardiac injury syndromes, a decrease in hemoglobin levels may be explained by active bleeding soon after the cardiac intervention, which leads to the accumulation of hemorrhagic fluid within the pericardial sac and potentially to cardiac tamponade. This aspect has been evaluated by Kramer et al^22^ in patients with post-pericardiotomy syndrome. They reported that the disruption of the pericardial membrane due to surgery led to contamination of the pericardial fluid with hemolyzed blood and high concentrations of oxidized hemoglobin,^22^ thus increasing local oxidative stress. Possibly the surgical injury can prime RBC hemolysis, recruitment of inflammatory cells, especially neutrophils and monocytes, that release pro-oxidative and proinflammatory molecules.^7^^,^^23^

Patients with idiopathic pericarditis were more likely to fail treatments and have recurrences as compared with subjects with postcardiac injury pericarditis. In this cohort, idiopathic pericarditis was more frequently diagnosed in black patients, and this is consistent with another report by Awan et al*.*^24^ The diagnosis of idiopathic pericarditis was less frequent in this cohort, as compared with what was found in previous studies conducted in Europe some years ago.^14^^,^^16^^,^^25^ The difference may be related to a recent increase in the number of procedures, such as atrial fibrillation ablation, pacemaker implantation, coronary artery bypass surgery, and transcatheter aortic valve implantation.^6^^,^^26^^,^^27^ Postcardiac injury pericarditis can be complicated by recurrences, cardiac tamponade, and constrictive evolution; however, the reported incidence is lower than that of idiopathic pericarditis.^28^ The etiopathogenesis of postcardiac injury pericarditis is not completely understood, but it is likely to be also an immune-mediated response to injuries deriving from the procedures. The initial pericardial injury secondary to ischemia (post-myocardial infarction pericarditis) or to physical or chemical trauma (pericarditis following cardiac procedures) may elicit an exaggerated inflammatory response in predisposed individuals.^6^

The higher observed incidence of treatment failure or pericarditis recurrence in patients with idiopathic forms may rely on the differences in underlying pathogenesis or in the time to antiinflammatory pharmacotherapy. A shorter time to treatment is expected for postcardiac injury syndromes possibly attributable to scheduled follow-ups that allow for early identification and subsequently early modulation of the inflammatory burden. In contrast, patients with idiopathic pericarditis develop the symptoms in the outpatient setting without ready access to clinicians and are therefore expected to have an extended time-to-diagnosis and antiinflammatory treatment. Additionally, although both idiopathic and postcardiac injury pericarditis can share a similar common inflammatory response,^6^^,^^29^ the molecular mechanisms initiating this response may not be the same, which highlights the need for investigations of tailored therapies.

Patients with elevation in troponin I levels were found to have a lower risk for the development of recurrences and of the adverse composite outcome. The detection of increased troponin I levels in patients with pericarditis is an expression of the concomitant involvement of the subepicardial myocardium in the inflammatory process as well as ECG abnormalities.^30^ In contrast to coronary artery disease, the elevation in troponin levels in the serum is not a negative prognostic marker in patients with pericarditis.^31^^,^^32^ Imazio and colleagues^14^ had previously described that recurrences were more frequent in cases with “isolated” pericarditis with normal troponin I levels, as compared with those with myopericarditis or perimyocarditis with elevated troponin I levels. The data presented herein confirm and enlarge previous findings as they expand to a multi-ethnic population of patients in United States, including a significant proportion of black patients. We also found that patients with abnormal troponin I levels more frequently presented with heart failure signs and symptoms, and yet they had more favorable outcomes in terms of pericarditis, than those without troponin I elevation. Myopericardial inflammatory syndromes, in which the inflammation involves both the pericardium and the myocardium, may have different clinical and prognostic characteristics compared with “isolated” pericarditis, and therefore tailored therapies may be required.^30^ In this view, troponin I level determination should be considered in every patient with a suspicion of pericarditis to correctly diagnose perimyocarditis, myopericarditis, and “isolated” pericarditis to help risk stratify the patients.^31^ Possibly elevated troponin I levels are simply a surrogate for a type of pericarditis or a cause of pericarditis associated with reduced recurrences (ie, postcardiac injury). Distinct ICD codes for myocarditis and pericarditis are currently used, and in this cohort we considered the I30 code for pericarditis and thoroughly reviewed to verify the correspondence between the ICD-10 code (I30) and the clinical diagnosis. Although we are well aware that it is unlikely to have missed cases of pericarditis, we may have missed some cases of pericarditis in which myocardium was secondarily involved. Pericarditis and myocarditis may have some overlapping aspects and represent diverse manifestations in the spectrum of the same disease.

Hospitalization for acute pericarditis is recommended for patients with poor prognostic characteristics (eg, subacute onset, fever, cardiac tamponade, large pericardial effusion, oral anticoagulation therapy),^1^ whereas low-risk cases may be safely managed in the outpatient setting.^33^ Although our cohort is relatively young, the high number of hospitalizations for the first episode of acute pericarditis may reflect the burden of comorbidities of the population served at this large urban hospital.

Surgical procedures related to the pericardial disease include pericardiocentesis, pericardial window, and pericardiectomy. Pericardiocentesis and pericardial window are necessary in case of cardiac tamponade or in near tamponade.^34^ Pericardiectomy, instead, is performed to treat constrictive pericarditis or as fourth-line therapy in patients with multiple recurrences unresponsive to medical treatments.^20^^,^^35^ Accordingly, pericardial surgical procedures were more frequent in the group of patients with adverse outcomes.

Several cases of death occurred in our cohort but none attributed specifically to pericarditis. Indeed, acute pericarditis is generally associated with a rather low disease-specific mortality, although patients with pericarditis appear to have an increased risk of in-hospital mortality.^4^ The mechanisms of this association are unexplored, because the data derive from large epidemiologic studies.^4^ Death is more likely to occur in patients affected by severe comorbidities, and this correlation may be explained by other underlying processes or procedure complications (ie, sepsis).^36^

### Study Strengths and Limitations

There are several strengths in our study. First of all, the study differs from other studies in the field given the diagnostic accuracy guaranteed by a chart-level review. We meticulously reviewed each case to confirm the diagnosis of pericarditis and only included those subjects meeting the 2015 ESC guideline criteria.^1^ This aspect is particularly important considering that recently ICD codes for pericarditis were found to be unreliable for pericarditis diagnosis.^37^ Second, we were able to longitudinally evaluate the natural history and the complications. Third, we studied a population that included a significant number of black patients who are underrepresented in other series, thus yielding a higher diversity compared with other previously reported populations.^14^^,^^24^^,^^28^ Some limitations, however, should be acknowledged. First, despite the use of unbiased tools for the initial cohort identification of cases, it remains a retrospective study with potential selection bias and ascertainment bias related to missing data (ie, biomarkers), therefore limiting our conclusions. Second, it is a single-center study, although conducted in a large hospital serving different groups of patients, whose population is characterized by an important burden of comorbidities and experienced a high rate of hospitalization, thus partially limiting the generalization of the results. Third, the follow-up was limited to approximately 6 months, and possibly some complications, particularly the development of constrictive pericarditis, were not captured, also because the electronic health records only documented events from the main hospital and in-network clinics. We only used the ICD-10 code I30 and the corresponding ICD-9 codes to retrieve the cases for this study, and we may have missed some cases of pericarditis with secondary involvement of the myocardium in the inflammatory process, thus limiting the conclusions that can be made on patients with acute pericarditis and troponin elevation. We did not perform any adjustment for multiple tests when comparing groups, because these analyses were only meant to unravel possible differences among patients’ groups. Finally, we were not able to record the exact time to event for all patients; therefore, we could not model a survival analysis accounting for different follow-up times.

## Conclusion

Acute pericarditis, although mostly a self-limiting disease, is associated with a clinically significant rate of complications. Acute idiopathic and postcardiac injury pericarditis should be considered as two different entities, with separate mechanisms driving the disease, different complication profiles, and different natural histories of the disease. The measurement of serum troponin I levels could be useful to recognize patients with elevated levels who are paradoxically at lower risk of failure of treatment, recurrences, constrictive pericarditis, tamponade, or death. However, how to tailor the treatment plan for those with abnormal troponin I values remains unclear. Further prospective studies are warranted to better define the time window and characteristic of subacute onset of pericarditis and may aid in identifying pathophysiological mechanisms of and targeted therapy for idiopathic and postcardiac injury pericarditis**.**

## Supplementary Data

e-Online Data
