# Supplementary material for: Clinical Presentation and Outcomes of Acute Pericarditis in a Large Urban Hospital in the United States of America
Source: Chest. 2020 Jul 24;158(6):2556–67. doi: 10.1016/j.chest.2020.07.039 (PMC7768931; doi:10.1016/j.chest.2020.07.039)
Supplement: e-Online Data [file mmc1.pdf]

CHEST Online Supplement

Clinical Presentation and Outcomes of Acute Pericarditis in a Large Urban Hospital in the United States of America

Alessandra Vecchié, MD; Juan G. Chiabrando, MD; Megan S. Dell, BSBME; Aldo Bonaventura, MD; Adolfo G. Mauro, PhD; George Wohlford, PharmD; Benjamin W. Van Tassell, PharmD; Daniel H. Berrocal, MD, PhD; Fabrizio Montecucco, MD, PhD; Anna Beutler, MD; John F. Paolini, MD, PhD; Tamas S. Gal, PhD; and Antonio Abbate, MD, PhD

CHEST 2020; 158(6):2556-2567

Online supplements are not copyedited prior to posting and the author(s) take full responsibility for the accuracy of all data.

© 2020 AMERICAN COLLEGE OF CHEST PHYSICIANS. Reproduction of this article is prohibited without written permission from the American College of Chest Physicians. See online for more details. **DOI:** 10.1016/j.chest.2020.07.039

e-Table 1. Characteristics of patients according to availability of troponin I determination.

	Patients without troponin I determination (n=73)	Patients with troponin I determination(n=167)	*p*-value
**Demographics Characteristics**			
Male sex	37 (50.7)	98 (58.7)	.261
Age, years	56 [35.0-68.5]	50 [33.0-61.0]	.173
Race			
Caucasian	49 (67.1)	72 (43.1)	**<.001**
Black or African-American	14 (19.2)	86 (51.5)	
Other	10 (13.6)	9 (5.4)	
**Medical History**			
Hypertension	33 (45.2)	93 (55.7)	.160
Diabetes	13 (17.8)	38 (22.8)	.493
Dyslipidemia	26 (35.6)	53 (31.7)	.554
Coronary artery disease	13 (17.8)	39 (23.4)	.396
Chronic heart failure	17 (23.3)	34 (20.4)	.611
Stroke/TIA	5 (6.8)	8 (4.8)	.542
Atrial fibrillation	34 (46.6)	29 (17.4)	**<.001**
Myocardial infarction	5 (6.8)	28 (16.8)	**.043**
Active smokers	10 (13.7)	67 (40.4)	**<.001**
Autoimmune diseases	6(8.2)	18 (10.8)	.645
Tuberculosis	0	2 (1.2)	1.000
Chest radiation	1 (1.4)	7 (4.2)	.441
Neoplastic diseases	5 (6.8)	25 (15.0)	.092
Severe chronic kidney disease	17 (23.3)	27 (16.3)	.208
Chest trauma	2 (2.7)	4 (2.4)	1.000
Recent cardiac procedure			
Percutaneous coronary intervention	1 (1.4)	6 (3.6)	**<.001**
Pacemaker/Cardiac ablation	19 (26.0)	5 (3.0)	
Cardiac Surgery	7 (9.6)	12 (7.2)	
**Therapies**			
Immunosuppression	4 (5.6)	11 (6.6)	1.000
High dose corticosteroids	28 (38.4)	98 (58.7)	.131
Oral anticoagulation	11 (15.5)	16 (9.6)	.189
**Etiology**			
Idiopathic	28 (38.4)	98 (58.7)	**<.001**
Post-cardiac injury	41 (56.2)	38 (22.8)	
Other	4 (5.6)	31 (18.6)	
**Clinical Presentation**			
Fever	3 (4.4)	18 (10.8)	.137
Subacute presentation	4 (5.6)	8 (4.8)	.754
Trauma	6 (8.5)	3 (1.8)	**.022**
Severe pericardial effusion	20 (28.2)	29 (17.4)	.079
Heart failure	11 (15.5)	32 (19.2)	.583
Chest pain	62 (89.9)	158 (94.6)	.253
**Cardiac Exam**			
Pericardial rub	12 (17.9)	31 (18.8)	1.000
Pulsus paradoxus	2 (3.0)	8 (4.8)	.728
Kussmaul sign	1 (1.5)	3 (1.8)	1.000

Online supplements are not copyedited prior to postinsting and the author(s)) take full responsibility for the accuracy of all data.

**EKG**			
PR depression	8 (12.3)	63 (38.2)	**<.001**
ST elevation	19 (29.2)	85 (51.5)	**.003**
T wave inversion	18 (27.7)	40 (24.2)	.615
**Laboratory**			
WBC, n x10$_3$/mL	8.6 [6.70-12.0]	9.6 [6.70-12.3]	.664
Hgb, g/dL	12.1 [10.55-13.50]	12.1 [10.4 -13.7]	.658
Platelets, n x10$_3$/mL	238 [179-342]	242 [10.4-13.7]	.964
Creatinine, mg/dL	0.83 [0.69-0.967]	0.93 [0.73-1.18]	**.029**
CRP, mg/L	0.8 [0 -3.6]	11.1 [2.05-18.9]	**.008**
ESR, mm/h	25 [3-72]	56 [29.5-92.75]	.161
**Treatment at Presentation**			
NSAIDs	44 (61.1)	125 (74.9)	**.044**
Colchicine	45 (62.5)	107 (64.1)	.884
Glucocorticoids	12 (16.9)	17 (10.2)	.193
Surgical Procedure			
Pericardiectomy	5 (6.9)	3 (1.8)	
Pericardial window	2 (2.8)	5 (3.0)	**.002**
Pericardiocentesis	15 (20.8)	12 (7.2)	
**Outcomes**			
Composite outcome*	33 (45.2)	29 (29.3)	**.019**
Failure of treatment	8 (11.4)	9 (5.4)	.107
Recurrent pericarditis	15 (20.5)	22 (13.2)	.174
Cardiac tamponade	13 (17.8)	18 (10.8)	.148
Constrictive pericarditis	6 (8.2)	3 (1.8)	**.025**
Death	2 (2.7)	7 (4.2)	.726
Rehospitalization due to any cause	29 (39.8)	61 (35.9)	.740

*Composite endpoint defined as the presence of any of the following: failure of treatment, recurrences, cardiac tamponade, constrictive pericarditis or death

The data are presented as a number and (%) of all cases or as median and [interquartile range].
P-values were calculated by Mann-Whitney U test or Fisher's exact test or c_2 test, as appropriate.
Statistical significant values are presented in bold character.

Abbreviations: CRP= C-reactive protein; EKG= electrocardiogram; ESR= erythro-sedimentation rate; Hgb= hemoglobin; NSAIDs= nonsteroidal anti-inflammatory drug; TIA= transient ischemic attack; WBC= white blood cells.

Online supplements are not copyedited prior to postinsting and the author(s)) take full responsibility for the accuracy of all data.

e-Table 2. Logistic regression model for the prediction of the composite outcome.

	Unadjusted		Adjusted	
**Composite adverse outcome**	**OR (95% CI)**	***p*-value**	**OR (95% CI)**	***p*-value**
Age	1.000 (0.986-1.015)	0.965		
**Sex**	**1.583 (0.925-2.710)**	**0.094**	1.111 (0.512-2.410)	0.789
BMI	1.004 (0.968-1.042)	0.822		
Ethnicity	0.839 (0.195-3.604)	0.813		
Race	1.047 (0.730-1.501)	0.804		
Etiological classification	0.914 (0.804-1.040)	0.173		
CHF	1.187 (0.624-2.259)	0.601		
**MI (history of)**	**0.383 (0.151-0.970)**	**0.043**	0.666 (0.201-2.161)	0.492
Chest radiation	1.162 (0.271-4.988)	0.840		
**Subacute onset**	**10.69 (2.28-50.07)**	**0.003**	**15.02 (1.852-[illegible]**	**0.013**
Immunosuppression	1.289 (0.443-3.757)	0.641		
High dose corticosteroids	2.468 (0.644-9.453)	0.187		
**Oral anticoagulation**	**0.209 (0.061-0.716)**	**0.013**	0.162 (0.020-1.334)	0.091
Heart failure at onset	1.158 (0.583-2.300)	0.675		
ESR	1.007 (0.996-1.018)	0.209		
CRP	1.039 (0.989-1.092)	0.124		
**Troponin>0.20 ng/mL**	**0.272 (0.107-0.693)**	**0.006**	**0.333 (0.119-[illegible]**	**0.035**
WBC	1.019 (0.960-1.081)	0.537		
**Hb**	**0.794 (0.700-0.901)**	**<0.001**	**0.753 (0.640-[illegible]**	**0.001**
NSAIDS	0.685 (0.384-1.222)	0.200		
**Colchicine**	**0.509 (0.293-0.883)**	**0.016**	0.911 (0.418-1.987)	0.815
Glucocorticoids	1.722 (0.784-3.784)	0.176		

Analysis performed for the composite endpoint defined as the presence of any of the following: failure of treatment, recurrences, cardiac tamponade, constrictive pericarditis or death.
Statistical significant values are presented in bold character.

Abbreviations: CI: confidence interval; OR: odd ratio.

e-Table 3. Characteristics of the cohort comparing idiopathic and post-cardiac injury etiologies.

	Idiopathic pericarditis (n=126)	Post-cardiac injury pericarditis (n=79)	*p*-value
**Demographic Characteristics**			
Male sex	68 (54.0)	46 (58.2)	.567
Age, years	48 [34-58]	61 [50-71]	**<.001**
Race			
Caucasian	49 (38.9)	55 (69.6)	
African American	70 (55.6)	14 (17.7)	**<.001**
Other	7 (5.6)	10 (12.7)	
**Medical History**			
Hypertension	64 (50.8)	46 (58.2)	.250
Diabetes	21 (16.7)	27 (34.2)	**.006**
Dyslipidemia	35 (27.8)	37 (46.8)	**.007**
Coronary artery disease	17 (13.5)	33 (41.8)	**<.001**
Congestive heart failure	15 (11.9)	28 (35.4)	**<.001**
Stroke/TIA*	5 (4.0)	7 (8.9)	.220
Atrial fibrillation	12 (9.5)	48 (60.8)	**<.001**
Myocardial infarction	7 (5.6)	22 (27.8)	**<.001**
Oral anticoagulation	4 (3.2)	19 (24.1)	**<.001**
**Clinical Presentation**			
Subacute presentation			.158
Severe pericardial effusion	23 (18.3)	17 (21.5)	.718
Heart failure	13 (10.3)	20 (25.3)	**.006**
Chest pain	116 (92.1)	72 (91.1)	.379
Fever	12 (9.5)	1 (1.3)	**.017**
**Laboratory**			
Troponin >0.20 ng/mL	9 (7.1)	22 (27.8)	**<.001**
**Outcomes**			
Composite outcome*****	48 (38.1)	20 (25.3)	.067
Failure of treatment	13 (10.3)	2 (2.5)	.051
Recurrent pericarditis	26 (20.6)	9 (11.4)	.126
Cardiac tamponade	13 (10.3)	9 (11.4)	.821
Constrictive pericarditis	7 (5.6)	1 (1.3)	.155
Death	5 (4.0)	2 (2.5)	.575
Failure or recurrences	33 (26.2)	10 (12.7)	**.022**
Rehospitalization due to any cause	47 (37.3)	33 (41.8)	.532

*Composite endpoint defined as the presence of any of the following: failure of treatment, recurrences, cardiac tamponade, constrictive pericarditis or death

The data are presented as a number and (%) of all cases or as median and [interquartile range].
P-values were calculated by Mann-Whitney U test or Fisher's exact test or c_2 test, as appropriate.
Statistical significant values are presented in bold character.

Abbreviations: TIA= transient ischemic attack.

Online supplements are not copyedited prior to postinsting and the author(s)) take full responsibility for the accuracy of all data.

e-Table 4. Characteristics of patients with abnormal serum troponin I levels (≥ 0.20 ng/mL).

	Troponin I < 0.20 ng/mL ng/ml (n=115)	Troponin I ≥ 0.20 ng/mL (n=52)	*p*-value
**Demographic Characteristics**			
Male sex	70 (57.9)	28 (60.9)	.861
Age, years	50 [36-60]	52 [31-62]	.920
Race			
Caucasian	50 (41.3)	22 (47.8)	
African American	66 (54.5)	20 (43.5)	.076
Other	5 (4.2)	4 (8.7)	
**Medical History**			
Hypertension	65 (53.7)	28 (60.9)	.486
Diabetes	28 (23.1)	10 (21.7)	1.000
Dyslipidemia	40 (33.1)	13 (28.3)	.583
Coronary artery disease	21 (17.4)	18 (39.1)	**.004**
Heart failure	20 (16.5)	14 (30.4)	.055
Stroke/TIA*	3 (2.5)	5 (10.9)	**.037**
Atrial fibrillation	17 (14)	12 (26.1)	.107
Myocardial infarction	11 (9.1)	17 (37)	**<.001**
Recent cardiac procedure			
Percutaneous coronary intervention	1 (0.8)	5 (10.9)	
Pacemaker/cardiac ablation	3 (2.5)	2 (4.3)	**.007**
Cardiac surgery	7 (5.8)	5 (10.9)	
**Therapies**			
Immunosuppression	7 (5.8)	4 (8.7)	.497
High dose corticosteroids	4 (3.3)	-	.576
Oral anticoagulation	9 (7.4)	7 (15.2)	.145
**Clinical Presentation**			
Subacute presentation	8 (6.6)	-	.108
Trauma	2 (1.7)	1 (2.2)	.576
Heart failure	18 (14.9)	14 (30.4)	**.028**
Severe pericardial effusion	24 (19.8)	5 (10.9)	.252
**Outcomes**			
Composite outcome *	43 (35.5)	6 (13)	**.004**
Failure to treat	9 (7.4)	-	.064
Recurrent pericarditis	20 (16.5)	2 (4.3)	**.041**
Cardiac tamponade	16 (13.2)	2 (4.3)	.160
Constrictive pericarditis	2 (1.7)	1 (2.2)	1.000
Death	5 (4.2)	2 (4.3)	1.000
Rehospitalization for any causes	44 (35.8)	18 (39.1)	.124

*Composite endpoint defined as the presence of any of the following: failure of treatment, recurrences, cardiac tamponade, constrictive pericarditis or death

The data are presented as a number and (%) of all cases or as median and [interquartile range].
P-values were calculated by Mann-Whitney U test or Fisher's exact test or c2 test, as appropriate.
Statistical significant values are presented in bold character.

Abbreviations: TIA= transient ischemic attack.

Online supplements are not copyedited prior to postinsting and the author(s)) take full responsibility for the accuracy of all data.

e-Figure 1. CONSORT diagram.

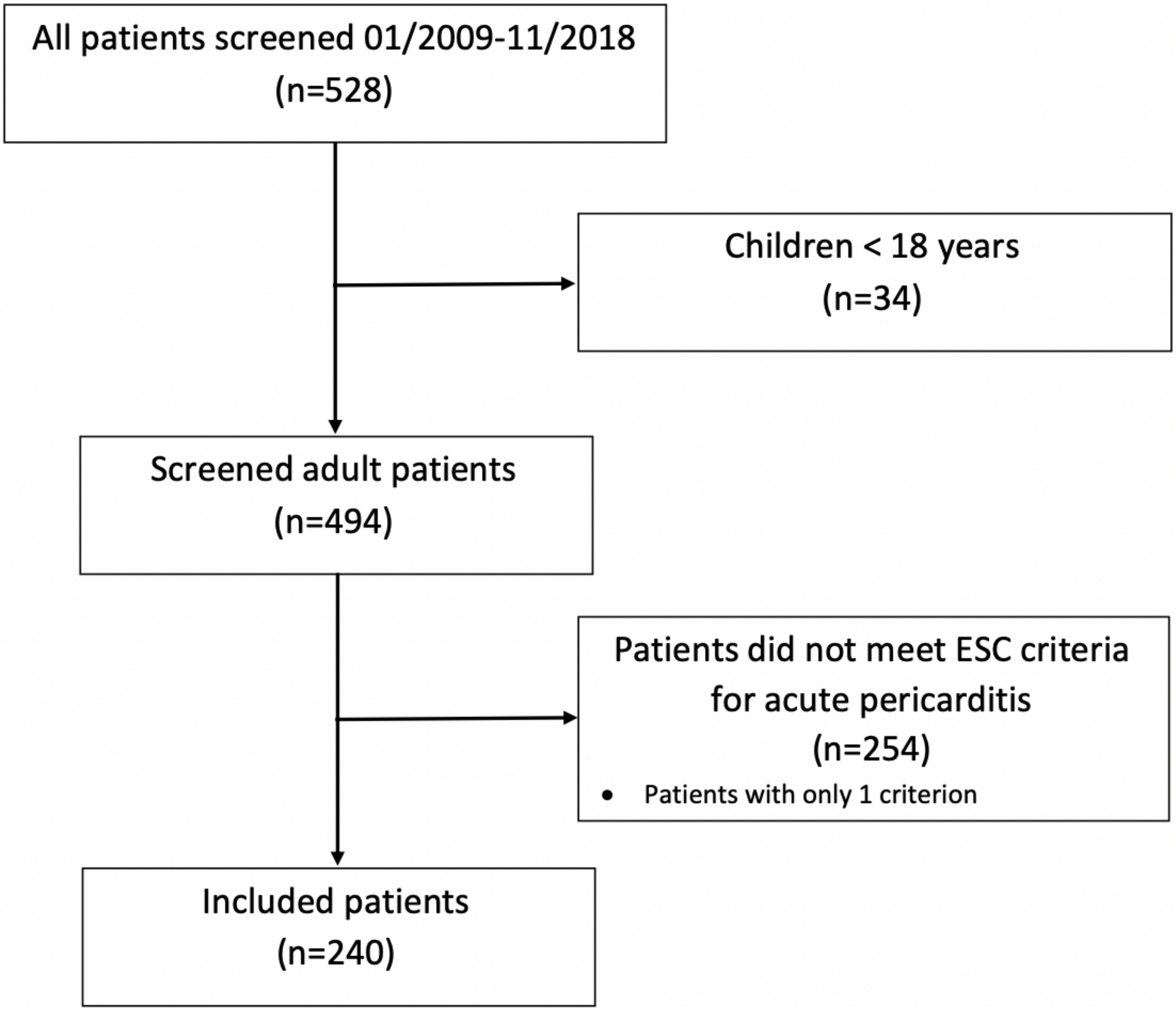

200285

Online supplements are not copyedited prior to postinsting and the author(s)) take full responsibility for the accuracy of all data.
